# Event and Comment

**Published:** 1907-07-15

**Authors:** 


### DENTAL REGISTER.

Vol. LXI. July 15, 1907. No. 7.

### EVENT AND COMMENT.

### Michigan Dental Association.

The annual convention of this Society was held this year at Saginaw, June 4th and 5th. The meeting was well though not as largely attended as the last two meetings. The local committee of arrangements did wonderfully well in providing a splended hall and clinic and exhibit rooms for the convention. The local entertainment too was all that could be desired, with the possible exception of an excess of rain for which the city could not well be held responsible. The meeting closed with a most delightful banquet and theatre party.

There were but three papers read, but they were on live topics and from original and enthusiastic sources, consequently the discussions were most valuable and interesting. The Subjects were Prophylaxis, by Dr. G. P. Rogers, Porcelain, by Dr. C. H. Land, and Oral Hygien in the public schools by Dr. George Zederbaum. We have seldom heard three so good papers in one meeting, and every one felt that the experiment of a few papers well considered was never more satisfactorily tried out.

The clinics were of unusual interest and many new as well as some old ideas were well presented.

Much time was consumed in adopting a new constitution and by-laws. The committee appointed at last year's convention to report on the Illinois scheme of reorganization, made a favorable report, and presented a new draft for a constitution, modeled on the Illinois plan, which was fully discussed and with some modifications adopted, and the new officers elected under this new constitution were instructed to complete the plan of redistricting the state, into local and district societies as soon as practicable. The new Board of Directors has just held a meeting and expects to have the scheme well under way during the coming year. By this means it is hoped to interest every ethical practitioner in the state in his local and consequently the state society

### Officers of Michigan Dental Association.

The officers for the ensuing year are; President, E. B. Spalding; Vice President, L. N. Hogarth; Secretary, George Copp; Treasurer, J. Ward House; and the following members of the Executive Council, C. H. Worboys, George Zederbaum, W. R. Purmort, J. W. Lyons, J. A. Armstrong, C. J. Bowles. The executive council has recently held a meeting and appointed committees which shall have charge of the various sections of the work of perfecting the organization of the state into local and district societies, and also in preparing programs for the meetings of the year. The next annual convention will be held the latter part of June and will consist of a boat ride to Sault Ste. Marie, and return The sessions will be held on the boat, Four days will be occupied on the trip, and it is planed that the social features will be the important business of the convention. It is proposed to invite the wives of the members, so that the occasion may be the more enjoyable. The boat will be chartered especially for the occasion, and the expense of transportation including meals and state room will not exceed eighteen dollars for the round trip. One of the large D. and C. boats has already been engaged. Such an event ought to bring out a large attendance and materially help to start the new method of increasing the effectiveness of the profession in a new and more perfect organization.

**Michigan's New Dental Law.**

The Michigan Legislature which has just adjourned passed a new dental law, which marks a new era in dental law making, to the extent that it defines specifically what constitutes a reputable dental college, and makes it obligatory on its Board of Examiners to pass upon all graduates upon a definite standard of Entrance requirement, as well as curriculum. The new law enacts substantially the standard set by the National Board of Examiners, which is a three years course of not less than 32 weeks of teaching and the requirements of a high school graduation for admission to the college. It also specifies that none but graduates of such schools can become candidates for examination. The old law compelled the Board to examine any one who applied whether a graduate or not, or whether he had ever attended a college a day or not. So far as we are aware Michigan is the first state to put into its statutes the standard of the National Board of Examiners; the Illinois Board has made it a rule of the Board. The new law also enacts a feasible and practical reciprocity clause which enables the Board to exchange registration of license with other states having an equivalent standard of registration. While this does not open the door for general reciprocity it makes possible reciprocity on a basis of equal standard, which is the only equitable basis upon which such affairs can be arranged. This law ought to be of general service because of the substantial support it gives the National Board of Examiners in its efforts to create a National and uniform standard.

